# Prognostic value of inflammatory markers for detecting anastomotic leakage after esophageal resection

**DOI:** 10.1186/s12893-020-00995-2

**Published:** 2020-12-09

**Authors:** Lukas F. Liesenfeld, Peter Sauer, Markus K. Diener, Ulf Hinz, Thomas Schmidt, Beat P. Müller-Stich, Thilo Hackert, Markus W. Büchler, Anja Schaible

**Affiliations:** 1 Department of Surgery, Heidelberg University Hospital, Im Neuenheimer Feld 420, 69120 Heidelberg, Germany; 2 Department of Gastroenterology, Heidelberg University Hospital, Im Neuenheimer Feld 410, 69120 Heidelberg, Germany

**Keywords:** Esophageal carcinoma, Esophageal resection, Anastomotic leakage, White blood cell count, C-reactive protein, Nun score

## Abstract

**Background:**

Early diagnosis of anastomotic leakage (AL) after esophageal resection is crucial for the successful management of this complication. Inflammatory serological markers are indicators of complications during the postoperative course. The aim of the present study was to evaluate the prognostic value of routine inflammatory markers to predict anastomotic leakage after transthoracic esophageal resection.

**Methods:**

Data from all consecutive patients undergoing transthoracic esophageal resection between January 2010 and December 2016 were analyzed from a prospective database. Besides clinicodemographic parameters, C-reactive protein, white blood cell count and albumin were analyzed and the Noble/Underwood (NUn) score was calculated to evaluate their predictive value for postoperative anastomotic leakage. Diagnostic accuracy was measured by sensitivity, specificity, and negative and positive predictive values using area under the receiver operator characteristics curve.

**Results:**

Overall, 233 patients with transthoracic esophageal resection were analyzed, 30-day mortality in this group was 3.4%. 57 patients (24.5%) suffered from AL, 176 patients were in the AL negative group. We found significant differences in WBCC, CRP and NUn scores between patients with and without AL, but the analyzed markers did not show an independent relevant prognostic value. For CRP levels below 155 mg/dl from POD3 to POD 7 the negative predictive value for absence of AI was > 80%. Highest diagnostic accuracy was detected for CRP levels on 4^th^ POD with a cut-off value of 145 mg/l reaching negative predictive value of 87%.

**Conclusions:**

In contrast to their prognostic value in other surgical procedures, CRP, WBCC and NUn score cannot be recommended as independent markers for the prediction of anastomotic leakage after transthoracic esophageal resection. CRP is an accurate negative predictive marker and discrimination of AL and no-AL may be helpful for postoperative clinical management.

*Trial registration* The study was approved by the local ethical committee (S635-2013).

## Background

Despite advancements in chemotherapy and radiotherapy, surgery remains the only curative treatment option for locally advanced esophageal carcinoma [1]. However, multimodality neoadjuvant regimens are also crucial as they can increase rates of R0 resections, pathological complete responses, and local tumor control, which are beneficial for improved overall survival [1]. Surgery for locally advanced esophageal carcinoma is performed according to tumor localization relative to the gastroesophageal junction and using any of the following approaches: transhiatal, transthoracic (Ivor Lewis procedure), or tri-incisional esophagectomy (McKeown procedure [1]).

Improvements in perioperative management and surgical technique have resulted in a steady decrease of postoperative mortality [2, 3]. However, recent nationwide data in the US showed a higher mortality rate of esophagectomy than that in most contemporary case series (7% vs. 1–4%) [4–7]. Further, morbidity continues to be as high as 50% [8, 9]. Among the postoperative complications, anastomotic leakage (AL) is the most fatal and frequent cause of postoperative mortality [10]. The incidence of AL following esophageal resection varies widely, but it has been reported to reach as high as 53% [10–20]. The variations in the leakage rates are also because of lack of a generally accepted, accurate definition of an AL [21, 22]. Further, a gold standard for diagnosing and managing leakage has not yet been established.

Early AL detection and treatment initiation are important to limit contamination and minimize sepsis and ultimately mitigate leakage-related mortality [23, 24]. Unrecognized leakage results in development of mediastinitis that is associated with high morbidity and mortality [25, 26]. Thus, early diagnosis of AL before the patients present with symptoms of mediastinitis such as confusion, fever, pain, or cardiorespiratory insufficiency, is crucial. Furthermore, early suspicion of AL may help to exclude patients from fast-track protocols and avoid early oral feeding. However, there is no standardized method for AL management after esophageal resection. Treatment is usually decided on the size of the leak, the degree of local contamination, and severity of the associated system response [27]. The ultimate modality for controlling leakage is surgery with esophageal diversion, but it also has a critical impact on the patient’s quality of life [28–32].

Various studies have attempted to identify AL prior to the onset of systemic changes in patient status using several molecular markers [18, 33–39] including white blood cell count (WBCC) and C-reactive protein (CRP). In particular, CRP is increasingly being studied as an early marker for postoperative complications [40]. CRP is an acute-phase protein produced by hepatocytes in response to pro-inflammatory cytokines [41]. CRP levels increase following surgery and commonly peak after 48 h then decrease thereafter in patients with an uncomplicated postoperative course. Several prospective studies and meta-analyses showed that CRP is a useful marker of AL after colorectal surgery [42, 43]. Meanwhile, the role of CRP in esophageal resection is less established. Some authors have reported a significant diagnostic value of CRP to detect AL. CRP values of 141 mg/l to 229 mg/l, particularly on postoperative day (POD) 3 and 4, have been reported to show a high diagnostic accuracy for AL, with an AUC of 0.71 to 0.86 (Table 1) [14, 16–18]. Park et al. showed that CRP has a high prognostic value for detecting AL in patients without neoadjuvant treatment prior to surgery [19].

**Table 1:** Literature review of the prognostic markers for detecting anastomotic leakage after esophageal surgery

Authors	Year	n	Complication	Technique	AL detection	Value (POD)	Cut-off	SN	SP	AUC
Deitmar [10]	2009	558	AL	Transthoracic	8th POD (range, 2–16)	CRP (2) WBCC (8)	135 mg/l 10.5/nl	> 80 > 70	n/a n/a	n/a n/a
Dutta [14]	2011	136	AL	Transhiatal, transthoracic	6th POD (range, 2–13)	CRP (3) CRP (4)	180 mg/l 180 mg/l	82 71	63 83	0.81 0.86
Van Genderen [16]	2011	63	AL, cardiorespiratory, infections	Transthoracic	n/a	CRP (1) CRP (2) CRP (3)	108.5 mg/l 175 mg/l 179 mg/l	82 85 89	50 53 50	0.74 0.78 0.75
Warschkow [12]	2011	210	AL, pneumonia, UTI, central line infection, and others	Transhiatal, transthoracic	7th POD (range, 4–11.3)	CRP (4) CRP (7)	141 mg/l 162 mg/l	78 56	70 89	0.77 0.81
Noble [34]	2012	258	AL	Transhiatal, transthoracic, cervical	7th POD (range, 5–15)	CRP (4) CRP (5) WBCC (5) NUn score	180 mg/l 189 mg/l 9/nl > 10	75 78 78 95	47 63 58 49	0.69 0.75 0.72 0.8
Tsujimoto [33]	2012	61	AL	Transthoracic	7th POD (range, 5–12)	SIRS (4)	SIRS criteria*	73	71	0.72
Hoeboer [18]	2015	45	AL	Transthoracic, transhiatal	n/a	CRP (3) ΔCRP (3–0) PCT (3)	229 mg/l Δ55 mg/l 0.35 ng/ml	71 80 67	84 80 80	0.78 0.82 0.86
Baker [35]	2015	100	AL	Transthoracic	n/a	WBCC (1 to 10)	> 12/nl	92	34	n/a
Edagawa [44]	2015	108	AL	n/a	8th POD	CRP (3)	8.62 mg/dl	n/a	n/a	n/a
Findlay [15]	2015	248	AL	Transthoracic	7th POD (range, 3–18)	NUn score WBCC (4)	> 10 6.89/nl	0 94	94 21	0.49 0.64
Gordon [17]	2016	145	AL	Transthoracic	6th POD (range, 4–10)	CRP (2) CRP (3) CRP (6)	209 mg/l 190 mg/l 154 mg/l	100 100 100	61 59 78	0.82 0.8 0.91
Park [19]	2017	201	AL	Transthoracic	n/a	CRP(NT−) (3) CRP(NT+) (3)	171 mg/l 164 mg/l	69 80	78 70	0.82 0.71
Paireder [20]	2017	258	AL	Transhiatal, transthoracic	9th POD (range, 1–23)	NUn score	> 10	45	74	0.59
Asti [36]	2017	243	AL	Transthoracic, cervical	6th POD (n/a)	CRP (5) WBCC (5) PCT (5)	83 mg/l 8.9/nl 0.38 ng/ml	89 59 78	61 84 71	0.82 0.69 0.75
Nilsson [39]	2018	462	AL	Transthoracic, cervical	n/a	CRP (3) CRP (4)	221 mg/l 203 mg/l	59 57	83 82	0.75 0.73
Gao [37]	2019	96	AL	Transthoracic	n/a	Prealbumin (5)	128 g/l	100	50	n/a
Bundred [38]	2020	382	AL	Transthoracic, cervical	8th POD (n/a)	NUn score	> 10	73	65	0.77
Liesenfeld (present study)	2020	233	AL	Transthoracic	7th POD (range, 1–30)	WBC (4) CRP (4) NUn score	8/nl 145 mg/l > 9	64 68 49	58 63 68	0.67 0.65 0.68

AL: anastomotic leakage; AUC: Area under the curve; NT(±): (with/without) neoadjuvant therapy; PCT: procalcitonin; POD: postoperative day; SIRS: systemic inflammatory response syndrome; SN: sensitivity; SP: specificity; UTI: urinary tract infection; WBCC: white blood cell count; CRP: c-reactive protein; NUn: Noble and Underwood; n/a: not available

Meanwhile, the diagnostic accuracy of WBCC for AL is yet to be evaluated [10, 15]. Noble and Underwood developed a score consisting of WBCC, CRP, and albumin values on the 4th POD (NUn score) to detect AL after upper gastrointestinal resections with esophageal anastomosis and reported a sensitivity > 95% [34]. The present study aimed to evaluate and validate the prognostic value of CRP level, WBCC, and NUn score in detecting AL in a well-defined cohort after transthoracic esophageal resection.

## Methods

### Study design and patients

This single-center retrospective study was approved by the local ethical committee of Medical Faculty Heidelberg. Need for written informed consent was waived owing to the retrospective nature of the study. All consecutive patients undergoing elective transthoracic esophageal resection at our institution between January 2010 and December 2016 were included in the study (Table 2). Data were collected from a prospectively maintained database. The patients were divided into two groups according to the absence (AL-negative) or presence (AL-positive) of AL (Table 2).

**Table 2:** Patient characteristics

	Total (%)	AL-negative	AL-positive	p value
N	233 (100%)	176 (75.5%)	57 (24.5%)	
Sex				0.299
Male	194 (83.26%)	144 (61.8%)	50 (21.5%)	
Female	39 (16.74%)	32 (13.7%)	7 (3%)	
Tumor type				
None	2 (0.9%)			
AEG I-III	170 (72.9%)			
SCC	60 (25.8%)			
NEC	1 (0.4%)			
Neoadjuvant treatment^a^	182 (78.11%)	136 (74.7%)	46 (25.3%)	0.71
Reconstruction				0.638
Gastric tube	229 (98.3%)			
Colon	4 (1.7%)			
Length of ICU stay [days]	7 ± 20.1	7 ± 14.2	19 ± 27.4	< 0.001
Length of hospitalization [days]	19 ± 24.6	17 ± 15.5	46 ± 30.7	< 0.001
30-day mortality	8 (3.4%)	2 (0.9%)	6 (2.5%)	0.005

AL: anastomotic leakage; AEG: adenocarcinoma of esophagogastric junction; SCC: squamous cell carcinoma; NEC: neuroendocrine carcinoma; ICU: intensive care unit

^a^Radio- and/or chemotherapy

### Surgical standard of esophageal resection

A combination of midline laparotomy and right thoracotomy was performed for esophageal resection combined with two-field lymphadenectomy. All patients had an immediate reconstruction using either tubularized gastric conduit or colon. Patients were usually extubated in the operating room and transferred to the intensive care unit. Patient management during the postoperative period was standardized. While only water and tea were allowed from days 1–4, oral intake was increased stepwise from day 5 onwards. Data recorded included demographics, tumor characteristics, type of reconstruction, postoperative complications and mortality.

### Assessment of AL

AL was suspected according to the presence of the following clinical signs or pathologic systemic response: fever, increased white blood cell count or CRP levels in the absence of pulmonary or urinary tract infection, development of organ failure, including respiratory or renal failure, sepsis, poor neurologic function, or gastrointestinal content within the pleural drains [27]. In these cases, AL was confirmed according to extravasation of oral contrast at computed tomography scan and/or visualization of anastomotic defect at upper gastrointestinal endoscopy and/or surgical exploration. For the endoscopic detection of AL, a flexible video endoscopy system (i.e., GIF H-180, Olympus, Tokyo, Japan) was used by an endoscopic specialist.

Serial routine blood samples were taken daily in the pre- and postoperative period. Inflammatory parameters (i.e., WBCC and CRP levels) were analyzed from the day of operation until the 7th POD. The albumin level on 4th POD was also acquired to calculate the NUn score. The NUn score was calculated using the following formula: NUn score = 11.3894 + (0.005 × CRP) + 0.186 × WBCC) − (0.174 × albumin).

### Statistical analysis

Postoperative inflammatory laboratory values were compared using Wilcoxon-Mann–Whitney test. P value < 0.05 was considered significant. Detection accuracy was determined using the area under the receiver operator characteristics curve (AUROCC) [45, 46]. It is a direct measure for diagnostic accuracy of a test, with an AUC > 0.7 being regarded as a clinically useful test [46] AUC was calculated using the trapezoidal rule, and an AUC of 0.7–0.8 was considered acceptable, 0.8–0.9 excellent, and > 0.9 outstanding [46]. The optimal cut-off value for WBCC, CRP level, and NUn score was calculated using the Youden index (*Youden index* = *Sensitivity* + *Specificity* *−* 1). Data is given as median ± standard deviation or mean with range.

## Results

### Patient characteristics

We evaluated 233 consecutive patients who underwent elective transthoracic esophageal resection at our institution between January 2010 and December 2016. The majority of the patients were male (n = 194, 83.3%). The patient and tumor characteristics, as well as the perioperative and intraoperative details, are shown in Table 2. Transthoracic esophageal resection was performed due to malignant disease in 99.1% of patients (72.9% adenocarcinoma, 25.8% squamous carcinoma, 0.4% neuroendocrine carcinoma). Overall, 182 patients (78.1%) received neoadjuvant treatment (chemo- and/or radiotherapy) prior to surgery. The AL-negative and the AL-positive groups included 176 (75.5%) and 57 (24.5%) patients, respectively. There were no significant differences in clinicodemographic characteristics between the two groups.

**Fig. 1:**
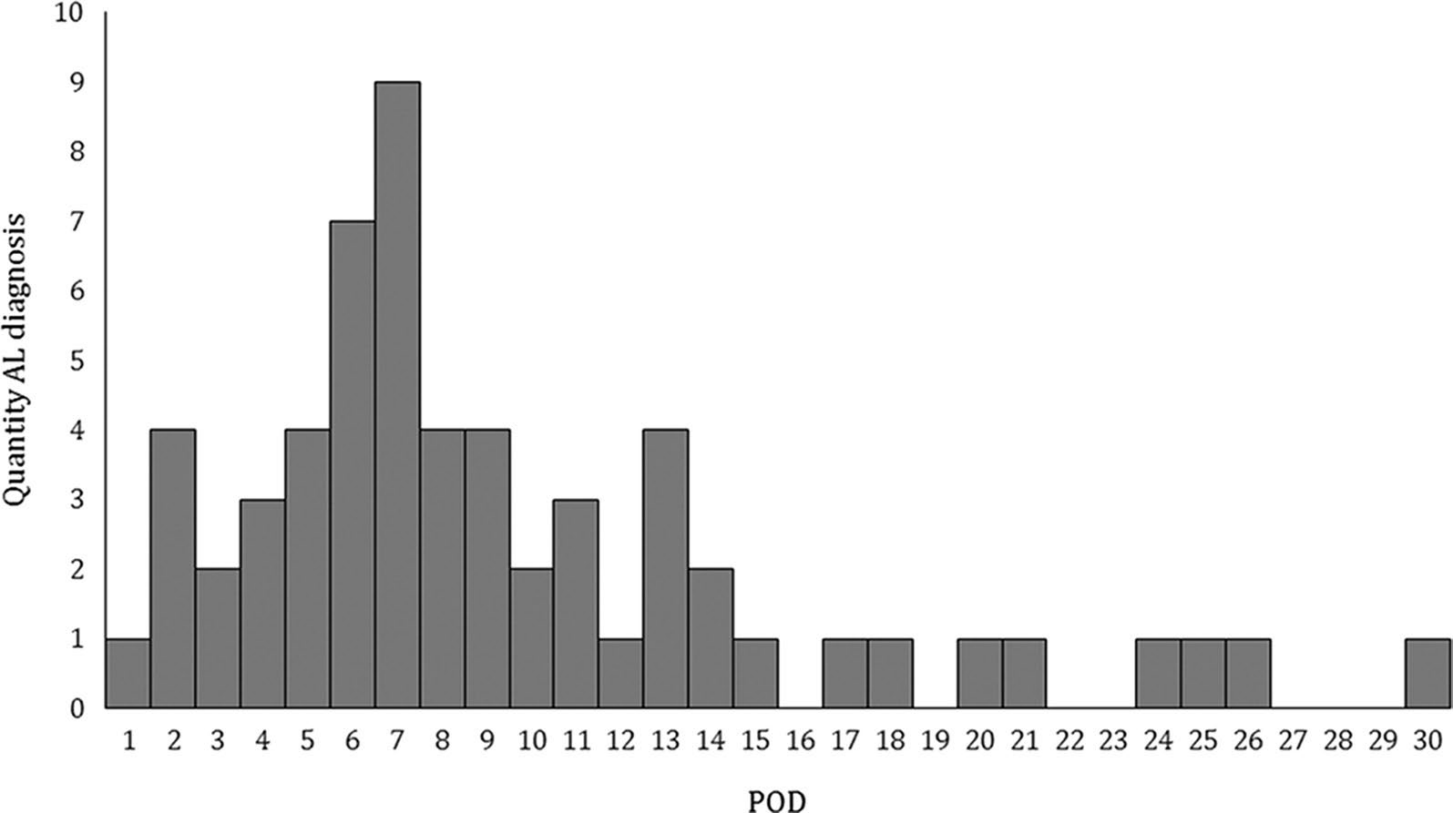
Histogram of quantity of AL by timepoint of diagnosis as postoperative day (POD). AL was most frequently diagnosed on 7th POD and overall approximately 50% were diagnosed prior 8th POD

Most of the patients (n = 229, 98.3%) underwent reconstruction with a gastric pull-up and intrathoracic anastomosis. Only 4 patients (1.7%) had a colonic interposition. The median time to AL diagnosis was 7 (± 6.4) days, and AL was diagnosed between the 1st to 30th POD (Fig. 1). For further analysis the 7 patients diagnosed with AL prior 4th POD were excluded due to assumed technical failure as source of AL. However, the withdrawal of these patients in the significances of the analyzed endpoints of the study showed no difference. There were no significant correlations between the occurrence of AL and sex (p = 0.3) and neoadjuvant therapy (p = 0.71). As expected, the length of intensive care unit stay (19 ± 27.4 days vs. 7 ± 14.2 days) and hospitalization (46 ± 30.7 days vs. 17 ± 15.5 days) were significantly higher in patients with AL (p < 0.001).

### WBCC

**Fig. 2:**
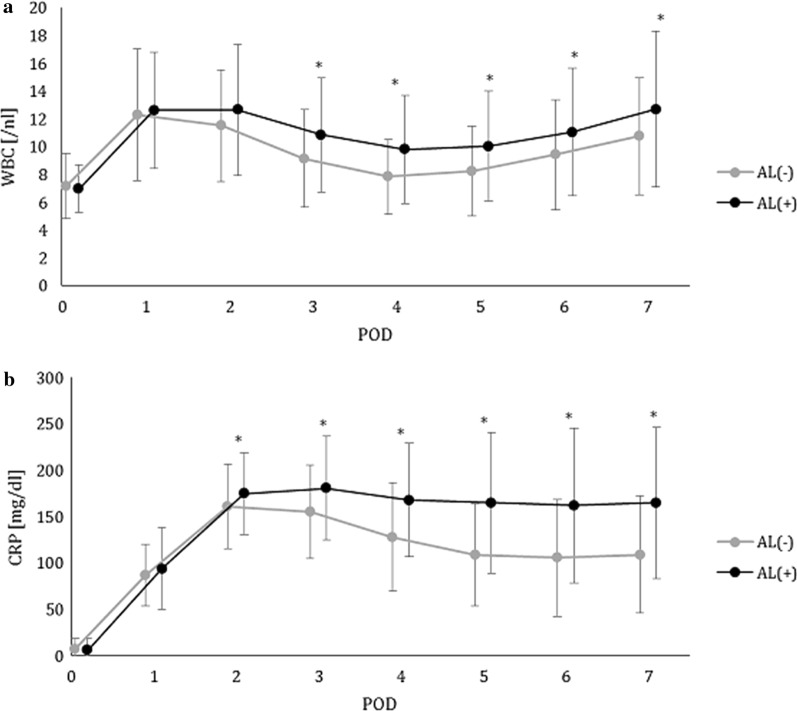
Pre- and postoperative WBCC (**a**) and CRP (**b**) values until the 7th POD after transthoracic esophageal resection. The black and grey lines represent patients with and without anastomotic leakage. Points represent mean values. Error bars show standard deviation. *p ≤ 0.05

**Fig. 3:**
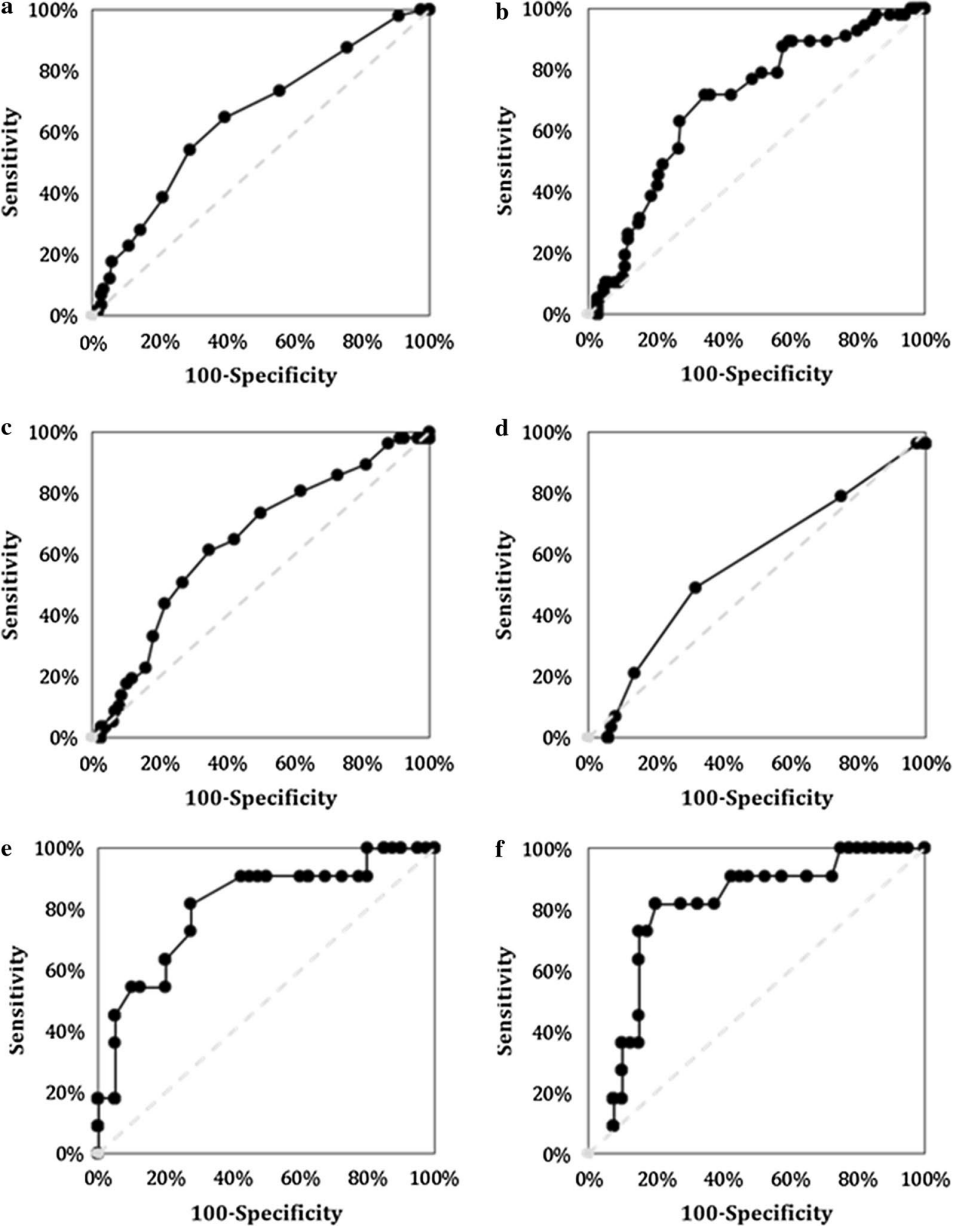
Receiver operating characteristic (ROC) curves of multivariate logistic regression of WBCC 4th POD (**a**), CRP 4th POD (**b**), ΔCRP 2nd–4th POD (**c**), NUn score (**d**), non-neoadjuvant CRP 4th (**e**), and 5th POD (**f**) for predicting anastomotic leakage after esophageal resection. True positive rate (sensitivity) is plotted in function of false-positive rate (100-specificity). The closer the graph to the upper left corner, the better the diagnostic accuracy. The area under the ROC curve (AUROCC) indicates the performance of the values for determining anastomotic leakage after esophageal resection. The AUC of WBCC 4th POD is 0.67 (p = 0.0005); CRP 4th POD, 0.65 (p < 0.0001); ΔCRP 2nd–4th POD, 0.62 (p = 0.0003); NUn score, 0.68 (p = 0.004); and non-neoadjuvant CRP 4th POD, 0.68 (p = 0.01) and 5th POD, 0.67 (p = 0.0009)

The mean WBCC level from the 1st to 7th POD in the AL-positive and the AL-negative group is shown in Fig. 2a. WBCC peaked on the 1st to 2nd POD, then consecutively decreased in both groups. However, the mean WBCC on the 3rd to 7th POD was significantly higher in the AL-positive group than that in the AL-negative group (range of means 9.8/nl to 12.7/nl vs.7.8/nl to 10.8/nl). The sensitivity and specificity rates of WBCC ranged from 44 to 65% and from 53 to 76%, respectively (Table 3). Best discrimination was possible on the 4th POD with a cut-off value of 8/nl; this had a negative predictive value (NPV) of 84.6% and a positive predictive value (PPV) of 31.3% (Fig. 3a). AUC values remained below 0.7. There were no significant differences in the absolute alterations of WBCC (Table 4).

**Table 3:** Diagnostic accuracy of WBCC and CRP for anastomotic leakage after transthoracic esophageal resection

WBCC	POD	AUC	Cut-off [/nl]	Sensitivity	Specificity	NPV	PPV	Accuracy	p value
	0	n/a	n/a	n/a	n/a	n/a	n/a	n/a	0.984
	1	n/a	n/a	n/a	n/a	n/a	n/a	n/a	0.337
	2	n/a	n/a	n/a	n/a	n/a	n/a	n/a	0.121
	3	0.65	11	44%	76%	82.1%	35.1%	0.71	0.005*
	4	0.67	8	64%	58%	84.6%	31.3%	0.62	0.0005*
	5	0.55	8	65%	53%	83.5%	28.9%	0.58	0.021*
	6	n/a	n/a	n/a	n/a	n/a	n/a	n/a	0.075
	7	n/a	n/a	n/a	n/a	n/a	n/a	n/a	0.246

CRP	POD	AUC	Cut-off [mg/l]	Sensitivity	Specificity	NPV	PPV	Accuracy	p value
	0	n/a	n/a	n/a	n/a	n/a	n/a	n/a	0.529
	1	n/a	n/a	n/a	n/a	n/a	n/a	n/a	0.363
	2	n/a	n/a	n/a	n/a	n/a	n/a	n/a	0.075
	3	0.6	150	72%	44%	84.3%	27.6%	0.52	0.014*
	4	0.65	145	68%	63%	86.9%	35.1%	0.91	0.0001*
	5	0.6	155	46%	71%	81.6%	31.7%	0.69	0.0001*
	6	0.53	120	64%	50%	82.4%	27.4%	0.57	0.0004*
	7	0.52	140	57%	51%	80%	25.5%	0.58	0.002*

WBCC: white blood cell count; CRP: C-reactive protein; NUn: Noble and Underwood; POD: postoperative day; AUC: Area under the curve; NPV: negative predictive value; PPV: positive predictive value; n/a: not available

* p ≤ 0.05

**Table 4:** Diagnostic accuracy of absolute alteration of WBCC and CRP for anastomotic leakage after transthoracic esophageal resection

WBCC	ΔPOD	AUC	Cut-off [/nl]	Sensitivity	Specificity	NPV	PPV	Accuracy	p value
	0 to 2	0.6	3	78%	36%	85%	26%	0.48	0.052
	2 to 3	0.67	n/a	n/a	n/a	n/a	n/a	n/a	0.17
	3 to 4	0.62	n/a	n/a	n/a	n/a	n/a	n/a	0.285
	4 to 5	0.5	n/a	n/a	n/a	n/a	n/a	n/a	0.121
	5 to 6	0.44	n/a	n/a	n/a	n/a	n/a	n/a	0.379

CRP	ΔPOD	AUC	Cut-off [mg/l]	Sensitivity (%)	Specificity (%)	NPV (%)	PPV (%)	Accuracy	p value
	1 to 3	0.57	90	48	63	80.5	27.9	0.64	0.011*
	2 to 3	0.59	0	54	61	81.7	29	0.64	0.024*
	2 to 4	0.62	− 20	5	63	83.5	31.6	0.66	0.0003*
	3 to 4	0.63	− 20	60	61	83.7	31	0.65	0.0001*
	2 to 5	0.59	− 50	68	51	84.3	29	0.59	0.00001*
	3 to 5	0.59	− 10	44	75	81.9	34.1	0.72	0.00008*

WBCC: white blood cell count; CRP: C-reactive protein; NUn: Noble and Underwood; ΔPOD: change of value (WBCC or CRP) between postoperative days; AUC: Area under the curve; NPV: negative predictive value; PPV: positive predictive value; n/a: not available

* p ≤ 0.05

Furthermore, we evaluated the absolute value and relative changes of WBCC in patients with AL 1 to 2 days prior diagnosis. WBCC on the day and 2 days before diagnosis showed a mean increase of 31% (range, − 28% to 186%) and 19% (range, − 19% to 187%), respectively. However, in 18.2% and 24% of the patients, the WBCC decreased 1 and 2 days prior to the diagnosis of AL. Evaluating WBCC in subpopulations with and without neoadjuvant treatment did not reveal better accuracy to discriminate AL (Additional file 1: Table S1 and Additional file 2: Table S2).

### CRP

The mean CRP level on the 1st to 7th POD for patients in the AL-positive and the AL-negative groups is shown in Fig. 2b. The CRP levels increased in both groups on the 1st and 2nd POD. However, while starting to decrease from the 3rd POD in the AL-negative group, CRP-levels remained high in the AL-positive group. There were significant differences in the CRP level from the 2nd to 7th POD between the two groups. The mean CRP values from the 2nd to 7th POD in the AL-negative group ranged from 105 mg/l to 161 mg/l and from 162 mg/l to 181 mg/l in the AL-positive group. The sensitivity and specificity rates ranged from 46 to 72% and from 44 to 71%, respectively (Table 3). Best discrimination was possible on the 4th POD with a cut-off value of 145 mg/l (AUC, 0.65; NPV, 86.9%; PPV, 35.1%; Table 3, Fig. 3b). There were significant differences in the absolute alterations of CRP (Table 4, Fig. 3c), but the sensitivity ranged from 44 to 68%, while the specificity ranged from 51 to 75%, with AUC values below 0.7.

Similar to the evaluation of WBCC, we also evaluated the absolute values and relative changes of CRP in patients with AL 1 to 2 days prior to diagnosis. The CRP showed a mean increase of 26% (range, − 20% to 138%) and 11% (range, − 30% to 119%) at 1 and 2 days before diagnosis, respectively. Nevertheless, the CRP values decreased in 38.1% and in 52% of the patients on 1 and 2 days prior to AL diagnosis. There were no significant differences in CRP according to the subgroups with and without neoadjuvant treatment (Additional file 1: Table S1 and Additional file 2: Table S2; Fig. 3e, f).

### NUn score

The mean NUn score was significantly different between the AL-negative and the AL-positive groups (8.6 mean (range, 5.2–12.1) vs 9.1 mean (range, 7.5–12.7); p = 0.006, Table 3). Depending on the cut-off values, the sensitivity rates ranged from 21 to 79%, while the specificity rates ranged from 25 to 86%, with an AUC of 0.68 (Fig. 3d). The optimal cut-off value of 10 recommended by Noble and Underwood had an NPV of 78.7% but only a PPV of 31.3%.

## Discussion

Patients who undergo esophageal resection are at risk of developing AL that consequently may lead to life-threatening mediastinitis and sepsis [25, 26, 47]. Therefore, early diagnosis of AL is crucial in further management and therapy [23, 24]. In colorectal surgery, the CRP level has been shown to be a useful early biomarker of AL [42, 48]. However, the role of CRP-levels as an indicator for AL after esophageal resection is unclear and preceding studies showed conflicting results. While some studies reported high diagnostic accuracy for CRP-levels predicting and detecting AL in the postoperative course [14, 17], others denied clinical relevance of these measures [34]. Comparably, the diagnostic accuracy of postoperative WBCC for AL remains unclear [10, 15]. In contrast to these two single parameters, the NUn score developed by Noble and Underwood has been reported to have high diagnostic accuracy for diagnosing AL after esophageal anastomosis [34]. In light of the mentioned disparity of preceding studies and the clinical relevance of potential early diagnostic markers for AL this monocentric study on 233 patients with esophageal resections aimed at reproducing diagnostic accuracy of CRP and WBCC levels, as well as the NUn score for detecting AL after esophageal resection.

We found significant differences in WBCC between patients with and without AL, but overall diagnostic accuracy did not reach clinical relevance. Although the general decrease of WBCC after the 2nd POD was less profound in patients with AL, optimal diagnostic accuracy was detected on the 4^th^ POD with a cut-off value of 8/nl, which is within the normal range of WBCC. Moreover, 20% of patients with AL showed also a decrease of WBCC 1 to 2 days prior to diagnosis, indicating missing relevance of these measures for the detection of AL following esophageal resection.

Similarly, we found significant differences in CRP between patients with and without AL with peaking CRP values on the 2nd POD in both groups and decreasing values on the 3rd POD only in the AL-negative group, but AUROCC analysis revealed low sensitivity and specificity rates as well as positive predictive values.

These findings are consistent with those in the literature where a significant difference in CRP level was noted between those with and without AL starting on the 2nd POD. This early difference in CRP was also observed by van Genderen et al. Hoeboer et al. and Gordon et al. [16–18]. In contrast, we could reproduce neither a relevant diagnostic accuracy for detecting AL nor a good PPV or cut-off-level in this study [12, 18]. Meanwhile, Park et al. reported that the CRP level has improved the diagnosis of AL in the subgroup of patients without neoadjuvant treatment [19]. In contrast, we again could not reproduce these findings in this collective. Analyzing relative percentage changes of CRP in subpopulations with and without neoadjuvant treatment did not reveal better results. One third of patients with AL showed a decrease of CRP 1 to 2 days prior diagnosis in our cohort, which also hampers clinical impact of these diagnostic measures. Therefore, CRP could not be identified as an reliable positive predictive marker and showed low specificity, which is consistent with the findings by Gordon et al. [17]. With respect to the NUn score, which was developed for the detection of AL after upper gastrointestinal surgeries, the reported cut-off values (> 10), high sensitivity (95%) and modest specificity (49%), could also not be reproduced in our study. [15, 20, 34].

In the present study, the CRP, WBCC, and NUn score did not achieve an AUC of > 0.7 and are therefore inadequate to be independent markers for detection of AL. Therefore, in contrast to lower GI surgery in patients with esophageal resection inflammatory markers are obviously more influenced by other complications such as pneumonia and cannot serve as independent markers. But we found CRP to be an accurate negative predictive marker: for CRP levels below 155 mg/dl from POD 3 to POD 7 the negative predictive value for absence of AL was > 80%. The best accuracy for CRP was on 4th POD with a cut-off value of 145 mg/l, which implies that 9 out of 10 patients with CRP level < 145 mg/l on the 4th POD do not have AL. Therefore, this study indicates that serum CRP concentration measured on the 4th to the 7th POD is a useful negative predictive marker for the development of AL, which may be helpful in postoperative patient management as these patients might be discharged earlier [17].

The retrospective nature of this study and variability in neoadjuvant treatments are indeed limitations of this study; otherwise, our cohort is homogenous, including only patients who underwent transthoracic esophageal resection. In order to improve detection and in sufficient time management of postoperative esophageal leakage, further prospective studies are needed to investigate new and better prognostic markers.

## Conclusions

The investigated inflammatory markers WBCC, CRP, and NUn score could not be identified as independent predictive markers of AL in the present study. But CRP values < 155 mg/l on the 3rd to 7th POD showed an acceptable performance as a negative predictor for AL and this discrimination of AL and no-AL maybe helpful for the postoperative clinical management.

## Supplementary Information

**Additional file 1: Table S1.** Diagnostic accuracy of WBCC and CRP for anastomotic leakage after transthoracic esophageal resection in patients without neoadjuvant treatment.

**Additional file 2: Table S2.** Diagnostic accuracy of WBCC and CRP for anastomotic leakage after transthoracic esophageal resection in patients with neoadjuvant treatment.

## Data Availability

The datasets generated and/or analyzed during the current study are not publicly available due to patient privacy and security of electronic medical information but are (anonymized) available from the corresponding author on reasonable request.

## Abbreviations

AL: Anastomotic leakage
AUC: Area under the curve
AUROCC: Area under the receiver operating characteristics curve
CRP: C-reactive protein
NPV: Negative predictive value
NUn: Noble and Underwood
POD: Postoperative day
PPV: Positive predictive value
WBCC: White blood cell count
