# Supplementary material for: Prognostic value of inflammatory markers for detecting anastomotic leakage after esophageal resection
Source: BMC Surg. 2020 Dec 9;20:324. doi: 10.1186/s12893-020-00995-2 (PMC7726907; doi:10.1186/s12893-020-00995-2)
Supplement: Supplementary file 1 — Additional file 1: Table S1. Diagnostic accuracy of WBCC and CRP for anastomotic leakage after transthoracic esophageal resection in patients without neoadjuvant treatment. [file 12893_2020_995_MOESM1_ESM.doc]

**Additional file 1: Table S1. Diagnostic accuracy of WBCC and CRP for anastomotic leakage after transthoracic esophageal resection in patients without neoadjuvant treatment**

| **WBCC** | **POD** | **AUC** | **Cut-off [/nl]** | **Sensitivity** | **Specificity** | **NPV** | **PPV** | **Accuracy** | **P value** |
| --- | --- | --- | --- | --- | --- | --- | --- | --- | --- |
|  | 0 | n/a | n/a | n/a | n/a | n/a | n/a | n/a | 0.423 |
|  | 1 | n/a | n/a | n/a | n/a | n/a | n/a | n/a | 0.748 |
|  | 2 | n/a | n/a | n/a | n/a | n/a | n/a | n/a | 0.645 |
|  | 3 | n/a | n/a | n/a | n/a | n/a | n/a | n/a | 0.429 |
|  | 4 | n/a | n/a | n/a | n/a | n/a | n/a | n/a | 0.068 |
|  | 5 | n/a | n/a | n/a | n/a | n/a | n/a | n/a | 0.267 |
|  | 6 | n/a | n/a | n/a | n/a | n/a | n/a | n/a | 0.528 |
|  | 7 | n/a | n/a | n/a | n/a | n/a | n/a | n/a | 0.177 |
| **CRP** |  |  | **Cut-off [mg/l]** |  |  |  |  |  |  |
|  | 0 | n/a | n/a | n/a | n/a | n/a | n/a | n/a | 0.483 |
|  | 1 | n/a | n/a | n/a | n/a | n/a | n/a | n/a | 0.928 |
|  | 2 | n/a | n/a | n/a | n/a | n/a | n/a | n/a | 0.149 |
|  | 3 | 0.64 | 210 | 50% | 76% | 83.6% | 37.7% | 0.73 | 0.03* |
|  | 4 | 0.68 | 150 | 78% | 62% | 90.4% | 37.5% | 0.69 | 0.01* |
|  | 5 | 0.67 | 160 | 78% | 65% | 90.9% | 39.8% | 0.68 | 0.0009* |
|  | 6 | 0.47 | 125 | 88% | 36% | 90.7% | 28.7% | 0.59 | 0.028* |
|  | 7 | n/a | n/a | n/a | n/a | n/a | n/a | n/a | 0.064 |

WBCC: white blood cell count; CRP: c-reactive protein; POD: postoperative day; AUC: Area under the curve; NPV: negative predictive value; PPV: positive predictive value; n/a: not available; *p≤0.05
