# Supplementary material for: Prognostic value of inflammatory markers for detecting anastomotic leakage after esophageal resection
Source: BMC Surg. 2020 Dec 9;20:324. doi: 10.1186/s12893-020-00995-2 (PMC7726907; doi:10.1186/s12893-020-00995-2)
Supplement: Supplementary file 2 — Additional file 2: Table S2. Diagnostic accuracy of WBCC and CRP for anastomotic leakage after transthoracic esophageal resection in patients with neoadjuvant treatment. [file 12893_2020_995_MOESM2_ESM.doc]

**Additional file 2: Table S2. Diagnostic accuracy of WBCC and CRP for anastomotic leakage after transthoracic esophageal resection in patients with neoadjuvant treatment**

| **WBCC** | **POD** | **AUC** | **Cut-off [/nl]** | **Sensitivity** | **Specificity** | **NPV** | **PPV** | **Accuracy** | **P value** |
| --- | --- | --- | --- | --- | --- | --- | --- | --- | --- |
|  | 0 | n/a | n/a | n/a | n/a | n/a | n/a | n/a | 0.624 |
|  | 1 | n/a | n/a | n/a | n/a | n/a | n/a | n/a | 0.549 |
|  | 2 | n/a | n/a | n/a | n/a | n/a | n/a | n/a | 0.085 |
|  | 3 | 0.68 | 11 | 46% | 81% | 83.6% | 41.7% | 0.65 | 0.007* |
|  | 4 | 0.7 | 8 | 61% | 62% | 84.3% | 32% | 0.61 | 0.002* |
|  | 5 | 0.58 | 8 | 62% | 58% | 83.6% | 30.3% | 0.53 | 0.03* |
|  | 6 | n/a | n/a | n/a | n/a | n/a | n/a | n/a | 0.114 |
|  | 7 | n/a | n/a | n/a | n/a | n/a | n/a | n/a | 0.726 |
| **CRP** |  |  | **Cut-off [mg/l]** |  |  |  |  |  |  |
|  | 0 | n/a | n/a | n/a | n/a | n/a | n/a | n/a | 0.242 |
|  | 1 | n/a | n/a | n/a | n/a | n/a | n/a | n/a | 0.484 |
|  | 2 | n/a | n/a | n/a | n/a | n/a | n/a | n/a | 0.112 |
|  | 3 | 0.61 | 150 | 71% | 46% | 84.3% | 28% | 0.51 | 0.044* |
|  | 4 | 0.66 | 145 | 63% | 68% | 86.2% | 36.7% | 0.63 | 0.002* |
|  | 5 | 0.59 | 85 | 82% | 36% | 87.2% | 27.5% | 0.45 | 0.001* |
|  | 6 | 0.58 | 85 | 76% | 42% | 85.4% | 27.8% | 0.43 | 0.001* |
|  | 7 | 0.48 | 140 | 57% | 59% | 82.1% | 28.9% | 0.51 | 0.019* |

WBCC: white blood cell count; CRP: c-reactive protein; POD: postoperative day; AUC: Area under the curve; NPV: negative predictive value; PPV: positive predictive value; n/a: not available; *p≤0.05
